# Using Structural Equation Modeling to Examine Pathways between Physical Activity and Sleep Quality among Chinese TikTok Users

**DOI:** 10.3390/ijerph19095142

**Published:** 2022-04-23

**Authors:** Xing Zhang, Siyuan Feng, Rui Peng, Hansen Li

**Affiliations:** 1 Department of Basketball and Volleyball, Chengdu Sport University, Chengdu 610041, China; starz-94@foxmail.com; 2 Laboratory of Genetics, University of Wisconsin-Madison, Madison, WI 53706, USA; siyuan.feng@wisc.edu; 3 Department of Chemistry, McGill University, Montreal, QC H3A 0B8, Canada; pengr_uestc@163.com; 4 Institute of Sports Science, College of Physical Education, Southwest University, Chongqing 400715, China

**Keywords:** TikTok, physical activity, sleep quality, mental health problems, structural equation model, mediation

## Abstract

TikTok, the most popular social media, brings various benefits to nowadays living. However, the problematic use of TikTok has also elicited a range of health problems, such as sleep problems. Physical activity (PA) appears to play a protective role in the problematic use of TikTok and its health consequences, but the pathways between PA and sleep health are understudied. Therefore, we aimed to propose a framework to check whether PA can benefit the sleep health of TikTok users by reducing bedtime delays for TikTok. Stress and mental health issues were also considered as they are potential mediators between PA and sleep health and may also influence the problematic use of smartphones. A cross-sectional investigation that involved 660 Chinese TikTok users was conducted in April 2021. The volume of PA, perceived stress (PSS-10), depression (PHQ-9), anxiety (GAD-7), bedtime delay for TikTok use, and sleep quality (PSQI) were investigated through an online questionnaire survey. Structural equation modeling was employed to examine pathways from PA to sleep quality through stress, mental health issues (depression and anxiety), and bedtime delay for TikTok. We found that PA exerted a significant effect on sleep quality through indirect pathways (β = −0.056, *p* = 0.001). Stress was a critical mediator of all indirect pathways, and the pathway mediated by stress and mental health issues made a major contribution to the total effect (β = −0.048, *p* = 0.002). The identified pathways mediated by bedtime delay for TikTok were relatively weak but significant. PA showed a distinct effect on bedtime delay for TikTok through stress and mental health issues (β = −0.043, *p* = 0.001). In conclusion, our framework highlights some pathways to understanding the benefits of PA on TikTok users’ sleep quality. Future research is warranted to explore extra indirect pathways and re-examine the causal relationships between variables.

## 1. Introduction

Sleep health is critical for daily living. Worldwide, more than 100 million people are suffering from sleep problems [1,2,3] due to diseases, emotional issues, and fast-changing schedules that disrupt normal circadian rhythms [4,5,6]. Nowadays, excessive social media usage is assumed to cause sleeping problems, especially in younger generations [7,8,9]. 

With the rise of short videos, a platform named TikTok provides convenient video editing tools for creators and offers viewers customized videos based on their preferences. Thus, TikTok has become extremely popular and attracted billions of users worldwide [10]. Some governments even employ TikTok to deliver public information [11]. Although TikTok contributes to social communication and entertainment, its dark side needs our attention. Some studies have suggested the impacts associated with the dark side, including eating disorders [12] and violence [12]. Addictive or problematic use of TikTok is a major concern [13,14], especially the usage at night, which has threatened the sleep health of the public [15].

Physical activity (PA) is a promising method to cope with the addictive or problematic use of smartphones [16,17], which may also help reduce the addictive or problematic use of TikTok. It is reported that adolescents with higher levels of physical activity tend to have lower stress, less problematic internet use, and better sleep [18], which indicates a possible role of daily PA in benefiting TikTok users. 

PA is well known to positively impact human health. Aside from the direct impact, previous studies on the general public have identified various mediators that contribute to indirect impacts. Based on the above clues, the problematic use of TikTok may also be a mediator that helps realize the indirect impacts. Therefore, the current study aimed to propose and examine a framework in which PA reduces the overuse of TikTok at nighttime and improves sleep conditions. This framework may help explore some pathways from PA to sleep health among TikTok users. Given the multiple potential mediators and their interaction, we tried to develop the framework based on the following clues.

## 2. Conceptual Framework

PA has a range of benefits on human health, including reducing stress, mental health issues, problematic behaviors, and sleep problems. Negative associations have been often observed between physical activity and stress [19]. Further, controlled trials have evidenced that PA can reduce stress [20], possibly by inhibiting the production of stress hormones [21]. Similar findings have also been obtained for anxiety and depression among children, adolescents, and adults [22,23], making PA a potential non-drug therapy for the two typical mental health issues [24]. PA may also reduce problematic behaviors such as smartphone addiction and drug abuse [16,25], which can be related to the regulation of the dopamine cycle [26]. Moreover, numerous studies have collectively shown that both acute and long-term exercise could benefit sleep quality [27,28].

Stress, anxiety, and depression are three highly related variables harmful to sleep health. Specifically, stress can lead to sleep disorder by activating the hypothalamic-pituitary-adrenal system [29]; depression was usually regarded as a risk factor for developing insomnia [30]; and anxiety symptom was found to prolong sleep onset latency and shorten the duration of total sleep time [31]. These clues may indicate the mediatory roles of stress, anxiety, and depression that link PA and sleep health. Besides, studies have explored their potential relationships. In a sense, anxiety and depression can be induced by stress, as stress can negatively influence medial prefrontal cortex functions and alter immune functions [32,33]. 

On the other hand, stress, anxiety, and depression are possible causes of problematic behaviors, such as problematic alcohol use [34]. Chiu [35] has reported that emotional stresses had positive predictive power for smartphone addiction. This may be because external factors like stress can contribute to the development of addictions [36,37,38]. Depression and anxiety may play similar roles. Some clues have also indicated that mental health issues are positively associated with problematic smartphone use [39]. It is assumed that mental health issues may decrease individuals’ ability to suppress and control undesired behaviors [16,40], which implies the negative role of mental health issues on problematic smartphone use. For these reasons, some studies have treated depression and anxiety as predictors of smartphone use [41,42]. A previous study has found that stress, depression, and anxiety are associated with problematic internet use, and coping stress was a reason for adolescents to use the internet [43]. It is assumed that escaping poor living conditions under stress is a reason for problematic internet usage [44]. 

Nowadays, over 99% of internet access is realized via smartphones in China, and 88.3% of the total internet usage was attributed to short video watching [45]. Therefore, the TikTok issue can be regarded as part of internet and smartphone issues. Like other users who resort to the internet and smartphone for escaping stressors in daily living, TikTok users may also watch short videos for stress reduction purposes. This recreation appears to have nothing harmful. However, overattachment to TikTok can be very common and negative, which appears to be a general problem of smartphone recreation [46]. Wang and Scherr [14] have highlighted that TikTok watching is a threat to the sleep health of Chinese users. Some previous problematic smartphone issues may also present for TikTok users, for example, bedtime delay [47,48]. Bedtime delay refers to the case that people automatically sleep later than they want. Bedtime delay for TikTok is regarded as a problematic and even addictive behavior that threatens sleep health [10,15].

**Figure 1:**
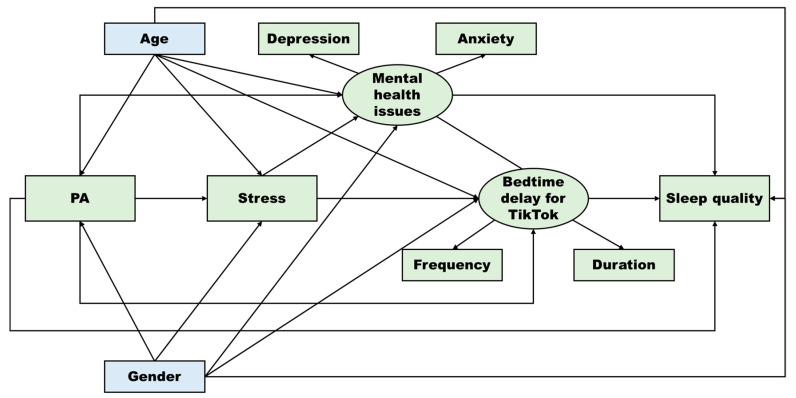
Conceptual model of physical activity–sleep quality pathways for TikTok users.

The role of PA in reducing the problematic use of smartphones has received more attention [16,17]. Park [18] reported that adolescents with higher levels of PA are likely to feel less stress, perform less problematic internet use, and have better sleep. These clues may indicate the chance of PA in reducing bedtime delay for TikTok and improving sleep quality. To explore the potential pathways linking PA, bedtime delay, and sleep quality, we developed the following framework (Figure 1) based on the above theoretical pathways between the core variables (PA, stress, mental health issues, bedtime delay for TikTok, sleep quality). Moreover, we also included gender and age as control variables because they may affect PA participation [49], sensitivity to stress and mental health issues [50,51], usage of social media or networks [52,53], and sleep quality [54,55].

## 3. Materials and Methods

### 3.1. Procedure and Participants

We conducted our cross-sectional investigation via "WeChat" from 20–22 April 2021. First, we recruited 50 students from Chengdu Sport University (n = 25) and Southwest University volunteers (n = 25) to help deliver our recruiting message through their known online groups. We also used a snowball method to kindly ask people who received our message to call for participation via their social circle. To encourage participation and maximize the response rate, we offered 8 RMB as an award for participation.

Participants were required to participate using a WeChat account linked to their personal legal identity, and their device and IP address were monitored so that every person could only participate once. Meanwhile, we employed the quality verification system empowered by Wenjuanxing Co., Ltd. (Chansha, China) to identify and eliminate questionnaires with random answers.

Eventually, 1148 participants signed the written consent and submitted the questionnaire. Thereafter, questionnaires with unfinished items (n = 135) and identical answers (n = 26) were excluded. Moreover, 327 volunteers who used TikTok less than 60 min a day in the most recent month were excluded. Eventually, 660 valid questionnaires were included in the analysis. The study was approved and supervised by the Ethics Review Board of Southwest University, China.

### 3.2. Measures

#### 3.2.1. Sleep Quality

Sleep quality was measured using a validated Chinese version of the Pittsburgh Sleep Quality Index (PSQI) [56]. This instrument contains 19 self-rated items grouped into seven quality domains: subjective sleep quality, sleep latency, sleep duration, habitual sleep efficiency, sleep disturbances, use of sleeping medication, and daytime dysfunction. Every domain has a score from 0 to 3. The total score of PSQI ranges from 0 to 21. A higher score indicates worse sleep quality. 

#### 3.2.2. Stress

Stress was measured using a validated Chinese version of the Perceived Stress Scale (PSS-10) [57]. The 10-item version (PSS-10) consists of six negative and four positive items. The negative element is intended to assess lack of control and negative affective reactions, while the positive aspect measures the ability to cope with existing stressors. Each item is rated on a five-point scale from 0 = never to 4 = very often, covering the preceding month.

#### 3.2.3. Depression

Depression was measured using a Chinese version of the Patient Health Questionnaire (PHQ-9) [58], and its reliability and validity have been validated in the general population [58,59]. The PHQ-9 consists of nine questions based on the nine DSM-IV criteria for a major depressive episode. Each of the questions asks patients to select the frequency of the depressive symptoms they experienced in the two weeks before survey administration. Scores for each item range from 0, not at all, to 3, nearly every day. Scores between 10 and 14 indicate a moderate level of depressive symptoms, scores between 15 and 19 indicate moderately severe depression, and scores 20 and above indicate severe major depression.

#### 3.2.4. Anxiety

Anxiety was measured using a validated Chinese version of Generalized Anxiety Disorder (GAD-7) [60]. The scale is a 7-item self-rating instrument. Each item described one of the typical symptoms of GAD and was evaluated by the frequency in which that symptom emerged over the last two weeks: 0 = Not at all, 1 = Several days, 2 = More than half the days, and 3 = Nearly every day.

#### 3.2.5. Physical Activity

Physical activity was measured by frequency and intensity and formulated as the following questions respectively: 

Frequency: “How often did you do physical activity last month?” A 7-point Likert was used to collect answers, where 1 = once per week or less, 7 = almost every day. 

Duration: "Describe the average duration of your physical activity in last month?” Answers were collected in 7 categories: 1 = less than 15 min, 2 = 15 to 30 min, 3 = 30 to 45 min, 4 = 45 to 60 min, 5 = 60 to 75 min, 6 = 75 to 90 min, and 7 = more than 90 min. 

The product of the frequency and duration, which ranged between 1 and 49, was treated as a variable to indicate the volume of PA [61].

#### 3.2.6. Bedtime Delay for TikTok

Bedtime delay for TikTok was evaluated via frequency and duration of the event [48], and the questions were as follow: 

Frequency: “In the last month, how often did your bedtime delay due to automatically using TikTok?” A 7-point Likert was used to collect answers, where 1 = never, 7 = almost every day.

Duration: “In the last month, how long did you usually bedtime delay for TikTok every time?” Answers were collected in 7 categories: 1 = less than 15 min, 2 = 15 to 30 min, 3 = 30 to 45 min, 4 = 45 to 60 min, 5 = 60 to 75 min, 6 = 75 to 90 min, and 7 = more than 90 min. 

### 3.3. Statistical Analysis

#### 3.3.1. Internal Reliabilities of Instruments

Internal reliabilities of the measuring questionnaires were analyzed with Cronbach’s alpha. Cronbach’s alpha greater than 0.70 was considered acceptable [62]. 

#### 3.3.2. Structural Equations Modeling

Structural equation modeling (SEM) was employed to examine the pathways of the conceptual model [63], and the maximum-likelihood method was applied for the asymptotically unbiased, consistent, and efficient estimators [64]. The direct and indirect effects among the conceptual model were analyzed via the bootstrap method (5000 times sampling and 95% confidence interval) [65]. The goodness of fitting was assessed by the following items: value of χ^2^/df < 3.00, goodness-of-fit index (GFI) > 0.90, comparative fix index (CFI) > 0.90, normed-fit index (NFI) > 0.90, Tucker-Lewis index (TLI) > 0.90, adjusted goodness of fit index (AGFI) > 0.90, Bollen’s incremental fit index (IFI) > 0.90, and root mean square error of approximation (RMSEA) < 0.08. The factor loading for latent variables of the conceptual model over 0.5 was considered acceptable.

We tested the initial model (M0) and observed acceptable model fits and factor loading values (Supplementary Table S1). After that, we removed pathways without at least a marginal statistical significance (*p* > 0.1) [66]. The modified model (M1) with fine model fits was selected as the final model.

#### 3.3.3. Sensitivity Analyses

We established competing models by re-specifying localized points of theoretical causalities based on the following two theories (Supplementary Figure S1):

(1) Model 2 (M2): Sleep problems may predict mental health issues;
(2) Model 3 (M3): A reciprocal relationship may present between sleep problems and mental health issues [67];
(3) Model 4 (M4): A reciprocal relationship may present between physical activity and stress [68].

To check if the final model could fit users who used TikTok at night or had delayed bedtime for TikTok, we conducted subgroup analyses that compared the models for nighttime users (M5) and bedtime delay users (M6) with that for all users. 

The Akaike information criterion (AIC) and Bayesian Information Criterion (BIC) were used for competing model selection [69].

All the statistical analyses were conducted in SPSS 25.0 and AMOS 20.0 software (SPSS Inc. Chicago, IL, USA).

## 4. Results

### 4.1. Validation of Measurements

The Cronbach’s alpha for sleep quality (PSQI), Stress (PSS-10), Depression (PHQ-9), and Anxiety (GAD-7) was 0.86, 0.80, 0.93, and 0.93 respectively, indicating ideal internal consistency (Table 1).

### 4.2. Characteristics of the Study Population

Three hundred and sixty-six (55.55%) males and two hundred and ninety-four (45.45%) females were involved in the current study. All the participants were users of TikTok (use TikTok at least 60 min a day in the most recent month), and 646 (97.88%) participants usually watch TikTok at night (Table 2). Besides, 568 (86.06%) participants usually delay their bedtime for watching TikTok.

### 4.3. The Final SEM Model

**Figure 2:**
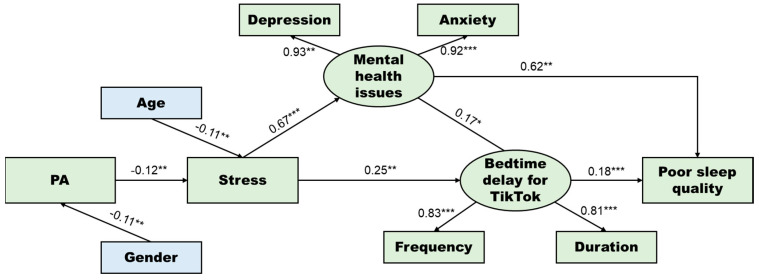
Final structural equation modeling (SEM) model describing the direct effects between variables. **Note:** * *p* < 0.05; ** *p* < 0.01; *** *p* < 0.001. As a higher Cronbach’s alpha for sleep quality (PSQI) score indicates worse sleep quality, the “sleep quality” in the framework was replaced with “poor sleep quality” in the final model.

As the initial model showed an acceptable fit to the data (χ^2^ = 28.575, *df* = 23, χ^2^/*df* = 1.242, *p* = 0.195, GFI = 0.991, AGFI =0.982, NFI = 0.987, IFI = 0.997, TLI = 0.996, CFI = 0.997, and RMSEA < 0.001), the factor loadings were ranged from 0.81 to 0.93, which were acceptable. The final model (M1) with direct pathways between variables is shown in Figure 2.

Table 3 demonstrates the standardized total, direct, and indirect effects of PA on core variables. PA showed significant total impacts on mental health issues (β = −0.079, *p* = 0.001), bedtime delay for TikTok (β = −0.043, *p* = 0.001), and poor sleep quality (β = −0.056, *p* = 0.001), and indirect pathways contributed to the total impacts.

The indirect pathways from PA to bedtime delay for TikTok or poor sleep quality are shown in Table 4. Two indirect pathways contributed to the total effect of PA on bedtime delay for TikTok, and the pathway mediated by bedtime delay and mental health contributed the most to the effect (β = −0.029, *p* = 0.001). Three pathways contributed to the total effect of PA on poor sleep quality, and the pathway mediated by stress and mental health issues contributed the most to the total effect (β = −0.048, *p* = 0.002). 

### 4.4. Sensitivity Analysis

Competing Model M2 (where poor sleep quality predicts mental health issues), Model M3 (where a reciprocal relationship presents between poor sleep quality and mental health issues), and Model M4 (where a reciprocal relationship presents between PA and stress) showed acceptable fits (Supplemental Figure S1 and Table S1). However, our final model (M1) retained the lowest AIC and BIC values, indicating a better fit than these competing models. We constrained the structural weights and compared the model specified for nighttime TikTok users only (M3) with that for total users (M1), and no significant difference was found between the two them (Δχ^2^ = 0.313, *df* = 6, *p* = 0.999). Likewise, no significant differences were observed between the model for bedtime delay users (M5) and the model for all users (M2) (Δχ^2^ = 8.573, *df* = 6, *p* = 0.199), indicating that the pathways in our framework can generally fit TikTok users.

## 5. Discussion

This study aims to develop a framework to explore pathways that link PA to the sleep quality of TikTok users. We found PA indirectly benefited sleep quality through stress, mental health issues, and bedtime delay for TikTok, which may underline the role of PA on human health in the short-video era. The indirect pathways mediated by stress and mental health are theoretically in line with the theories on the general public. The pathways mediated by bedtime delay for TikTok were not strong but significant. We found a distinctive impact of PA on bedtime delay for TikTok, which may indicate a special benefit of PA on TikTok users. The following discussion was focused on the pathways involved with bedtime delay for TikTok.

### 5.1. General Discussion

In the current study, stress was found to directly increase bedtime delay for TikTok, meanwhile indirectly increasing bedtime delay via increasing mental health problems. From a positive perspective, using TikTok could be a beneficial behavior. According to the Compensatory Internet Use Theory, people connect to the global network to avoid real-life problems and compensate for dysphoric moods [70]. Likewise, TikTok is an extended network and the most welcomed entertainment, which provides a solution for urban dwellers to regulate native emotions and avoid stressful conditions in urban living. However, as the compensation effect is found to correlate to negative emotions, the chronic stressful conditions in cities may lead to unhealthy attachments, such as known unhealthy attachments to social media, smartphones, and the internet [71,72,73]. These harmful conditions may partially explain the positive relationship observed between stress and bedtime delay for TikTok [74]. Besides, stress is known as an important cause of anxiety and depression. An identified pathway from stress to mental health problems also confirms this relationship [75,76,77]. These mental health problems are reported to limit individuals’ ability to suppress and control undesired behaviors, which may further lead to addictive behaviors, such as overuse of TikTok at night [16,40]. These findings may also explain the distinctive pathway from stress to bedtime delay via mental health problems.

Regarding the pathways from PA to sleep quality, sleep quality was not directly affected by PA. Instead, stress played an important mediatory role in contributing to all the distinctive pathways heading towards sleep quality. Specifically, PA has promoted sleep quality by relieving stress that led to mental health problems or bedtime delays for TikTok. In previous studies, PA promoted sleep quality in various populations [28,78,79,80]. Sleep quality benefits could be explained by positive mood changes, mental conditions, and autonomic nervous functions [81,82,83]. However, most existing studies only focused on outcomes or direct effects, while other indirect pathways are not very clear. Similar to our conceptual framework, Amer [84] observed significant correlations between PA, mental health problems, and sleep quality, but no causality was proposed. By comparison, our findings demonstrate a potential mechanism for the benefits of PA on sleep quality in TikTok users and underline the role of stress in increasing mental health problems. PA’s stress reduction function can be due to its positive impacts on hormones and neurotransmitters, such as dopamine and serotonin, which may benefit emotional outcomes and further enhance sleep quality [81,82,83,85]. 

On the other hand, sleep quality was not directly driven by stress either. Mental health problems and bedtime delay were found to be two mediators linking stress and sleep quality. Mental health problems have been proved to reduce sleep quality by decreasing sleep efficiency and increasing sleep onset latency [86,87]. In response, PA can effectively treat mental health problems by regulating the release and synthesis of brain-derived neurotrophic factors and the insulin-like growth factor, which may contribute to better sleep quality [88]. In terms of bedtime delay for TikTok, as we discussed above, it could be a stress-induced addictive behavior. Based on the design of TikTok, the short videos recommended based on users’ preferences may increase dopamine and lead to mental pleasure. Furthermore, a short video usually lasts merely several seconds, which improves users’ operations on TikTok compared to other media platforms. Thus, this TikTok design can lead to a more frequent stimulus-feedback-reward and become more addictive. Besides, the upcoming short videos are concealed for TikTok. The unexpected reward generated by uncovering a new short video may increase the activity of dopamine neurons, thus giving a positive feedback signal to the brain regions associated with the preceding behavior [89]. Apart from the subjective wishes of watching short videos at night, other known harmful factors of smartphones may also prolong bedtime delay by reducing sleep drive. For instance, the ruminative thoughts and emotional involvement activated by the nighttime use of smartphones can make users sleepless [7,90]. Meanwhile, the electromagnetic radiation and LED light of electronic equipment can inhibit melatonin (a hormone in the human body that induces natural sleep) and may further aggravate bedtime delay [91,92].

### 5.2. Limitation

Several limitations need to be considered when interpreting the results of the current study. 

First, the cross-sectional feature of the data may limit us from verifying the direction of causalities. That is an inherent limitation of all SEM methods using cross-sectional data. Although we have established several competing models based on extra theories, the issues of causality cannot be eliminated. Future studies may need to consider the time sequence of variables and may design several stages for data collecting. For example, measuring PA engagement of daytime and TikTok use on the same day night may help to support temporal precedence.

Second, we have taken some procedures to reduce common method variance (CMV), such as using concise well-studied instruments, excluding questionnaires with identical and random answers. However, CMV cannot be ruled out because we used a single investigation on the same participants to measure dependent and independent variables without a design for time intervals. Moreover, participants might not want to disclose their poor mental conditions and offered ambivalent answers. These may have inflated the relationships between the variables.

Third, most of our participants were young and middle-aged people. Only 0.15% of people aged over 69 years participated in our investigation, which is not consistent with the age structure of the general population of China (13.5% of China’s total population is over 65 years old). This is because we used a convenient method to recruit participants. The snowball method was likely to include college students and relevant people. Therefore, a single-informant bias must have been presented in our study. Moreover, we only employed self-reported measures, which may increase the informant bias. Future studies may need to employ stratified sampling methods and objective measures to obtain results that can be better generalized to the public.

## 6. Conclusions

The current study aimed to explore the role of PA on the problematic use of TikTok before sleep and the subsequent sleep problems. We found PA indirectly and positively impacted the sleep quality of TikTok users through stress, mental health issues, and bedtime delay for TikTok. These findings collectively support the positive role of PA in human behaviors and health. Moreover, we found several indirect pathways that negatively linked PA and bedtime delay for TikTok, which offers a framework to partially explain the known negative association between PA and problematic use of smartphones or the internet. Based on our findings, encouraging PA is a helpful method to benefit users of TikTok and maybe other short-video platforms. Nevertheless, it is noteworthy that our modeling was based on cross-sectional data, so the actual causal relationships between variables still require longitudinal controlled trials to re-examine.

## Figures and Tables

**Table 1:** Internal consistency of the questionnaires.

-	PSQI	PSS-10	PHQ-9	GAD-7
Dimension	Sleep quality	Stress	Depression	Anxiety
Cronbach’s α	0.86	0.80	0.93	0.92

**Table 2:** Summary statistics of the study population.

Variables	Category	Mean (SD)	Percentage
Gender	Male	-	55.45%
-	Female	-	44.55%
Age (years)	18<	-	5.45%
-	18~45	-	90.30%
-	46~69	-	4.09%
-	>69	-	0.15%
User of TikTok	-	-	100.00%
Watch TikTok at night	-	-	97.88%
Bedtime delay for TikTok	-	-	86.06%
Physical activity	Frequency (points)	3.49 (1.81)	-
-	Duration (score)	2.64 (1.25)	-
Bedtime delay for TikTok	Frequency (score)	3.07 (1.88)	-
-	Duration (score)	2.38 (1.60)	-
Stress (PSS-10 score)	-	15.30 (5.96)	-
Depression (PHQ-9 score)	-	4.48 (5.15)	-
Anxiety (GAD-7 score)	-	3.52 (3.94)	-
Sleep quality (PSQI score)	-	5.21 (3.22)	-

**Table 3:** Standardized total, direct, and indirect effects of physical activity (PA) on core variables.

Variable	Total β (95% CI)	*p*	Direct β (95% CI)	*p*	Indirect β (95% CI)	*p*
Stress	−0.118 (−0.194, −0.047)	0.001	−0.118 (−0.194, −0.047)	0.001	-	-
Mental health issues	−0.079 (−0.131, −0.032)	0.001	-	-	−0.079 (−0.131, −0.032)	0.001
Bedtime delay for TikTok	−0.043 (−0.075, −0.017)	0.001	-	-	−0.043 (−0.075, −0.017)	0.001
Poor sleep quality	−0.056 (−0.095, −0.023)	0.001	-	-	−0.056 (−0.095, −0.023)	0.001

**Note:** β, standardized effects.

**Table 4:** Indirect pathways from PA to bedtime delay for TikTok and poor sleep quality.

Pathways	β (95% CI)	*p*
PA → Stress → Bedtime delay	−0.013 (−0.033, −0.003)	*p =* 0.011
PA → Stress → Mental health issues → Bedtime delay	−0.029 (−0.059, −0.011)	*p =* 0.001
PA → Stress → Bedtime delay → Poor sleep quality	−0.005 (−0.012, −0.002)	*p* = 0.002
PA → Stress → Mental health issues → Poor sleep quality	−0.048 (−0.083, −0.018)	*p* = 0.002
PA → Stress → Mental health issues → Bedtime delay → Poor sleep quality	−0.002 (−0.006, −0.001)	*p* = 0.002

**Note:** PA, physical activity; bedtime delay, bedtime delay for TikTok.

## Data Availability

The data is available upon request from the corresponding author.

## Supplementary Materials

The following supporting information can be downloaded at: https://www.mdpi.com/article/10.3390/ijerph19095142/s1, Figure S1: Structures of competing models; Table S1: Model fits of the models.

## References

[1] Chattu V.K., Manzar M.D., Kumary S., Burman D., Spence D.W., Pandi-Perumal S.R. (2018). The Global Problem of Insufficient Sleep and Its Serious Public Health Implications. Healthcare.

[2] Ruiz-Castell M., Makovski T.T., Bocquet V., Stranges S. (2019). Sleep duration and multimorbidity in Luxembourg: Results from the European Health Examination Survey in Luxembourg, 2013–2015. BMJ Open.

[3] Stranges S., Dorn J.M., Shipley M.J., Kandala N.-B., Trevisan M., Miller M.A., Donahue R.P., Hovey K.M., Ferrie J.E., Marmot M.G. (2008). Correlates of Short and Long Sleep Duration: A Cross-Cultural Comparison Between the United Kingdom and the United States: The Whitehall II Study and the Western New York Health Study. Am. J. Epidemiol..

[4] Allen K.D., Renner J.B., Devellis B., Helmick C.G., Jordan J.M. (2008). Osteoarthritis and sleep: The Johnston County Osteoarthritis Project. J. Rheumatol..

[5] Palmer C.A., Oosterhoff B., Bower J.L., Kaplow J.B., Alfano C.A. (2018). Associations among adolescent sleep problems, emotion regulation, and affective disorders: Findings from a nationally representative sample. J. Psychiatr. Res..

[6] Billings J., Focht W. (2016). Firefighter Shift Schedules Affect Sleep Quality. J. Occup. Environ. Med..

[7] Woods H.C., Scott H. (2016). #Sleepyteens: Social media use in adolescence is associated with poor sleep quality, anxiety, depression and low self-esteem. J. Adolesc..

[8] Garett R., Liu S., Young S.D. (2018). The relationship between social media use and sleep quality among undergraduate students. Inf. Commun. Soc..

[9] Levenson J.C., Shensa A., Sidani J.E., Colditz J.B., Primack B.A. (2017). Social Media Use Before Bed and Sleep Disturbance among Young Adults in the United States: A Nationally Representative Study. Sleep.

[10] Zhang X., Wu Y., Liu S. (2019). Exploring short-form video application addiction: Socio-technical and attachment perspectives. Telemat. Inform..

[11] COVID-19 on TikTok: Harnessing an Emerging Social Media Platform to Convey Important Public Health Messages.

[12] Espel-Huynh H., Lewis-Smith H., Telléus G.K. Urgent Responsibility to Reduce Harms Posed by Social Media on risk for Eating Disorders: An Open Letter to Facebook, Instagram, TikTok, and Other Global Social Media Corporations. https://www.newswise.com/articles/urgent-responsibility-to-reduce-harms-posed-by-social-media-on-risk-for-eating-disorders.

[13] Yang Z., Griffiths M., Yan Z., Xu W. (2021). Can Watching Online Videos Be Addictive? A Qualitative Exploration of Online Video Watching among Chinese Young Adults. Int. J. Environ. Res. Public Heal..

[14] Wang K., Scherr S. (2021). Dance the Night Away: How Automatic TikTok Use Creates Pre-Sleep Cognitive Arousal and Daytime Fatigue. Mob. Media Commun..

[15] Wei H., Yi-he J., Qiong W., Nan W. (2021). Relationship between Short-form Video Social Media Addiction and Sleep Disturbance of College Students: The Mediating Role of Nighttime Social Media Use and the Moderating Role of Gender. Chin. J. Clin. Psychol..

[16] Liu S., Xiao T., Yang L., Loprinzi P.D. (2019). Exercise as an Alternative Approach for Treating Smartphone Addiction: A Systematic Review and Meta-Analysis of Random Controlled Trials. Int. J. Environ. Res. Public Health.

[17] Kim H. (2013). Exercise rehabilitation for smartphone addiction. J. Exerc. Rehabil..

[18] Park S. (2014). Associations of physical activity with sleep satisfaction, perceived stress, and problematic Internet use in Korean adolescents. BMC Public Health.

[19] Nguyen-Michel S.T., Unger J.B., Hamilton J., Spruijt-Metz D. (2006). Associations between physical activity and perceived stress/hassles in college students. Stress Health.

[20] van der Zwan J.E., de Vente W., Huizink A.C., Bögels S.M., de Bruin E.I. (2015). Physical Activity, Mindfulness Meditation, or Heart Rate Variability Biofeedback for Stress Reduction: A Randomized Controlled Trial. Appl. Psychophysiol. Biofeedback.

[21] Sallis J.F., Owen N. (1998). Physical Activity and Behavioral Medicine.

[22] Biddle S.J.H., Asare M. (2011). Physical activity and mental health in children and adolescents: A review of reviews. Br. J. Sports Med..

[23] Rebar A.L., Stanton R., Geard D., Short C., Duncan M.J., Vandelanotte C. (2015). A meta-meta-analysis of the effect of physical activity on depression and anxiety in non-clinical adult populations. Health Psychol. Rev..

[24] Ströhle A. (2008). Physical activity, exercise, depression and anxiety disorders. J. Neural Transm..

[25] Bardo M.T., Compton W.M. (2015). Does physical activity protect against drug abuse vulnerability?. Drug Alcohol Depend..

[26] Archer T., Badgaiyan R.D., Blum K. (2017). Physical Exercise Interventions for Drug Addictive Disorders. J. Reward Defic. Syndr. Addict. Sci..

[27] Wang F., Boros S. (2021). The effect of physical activity on sleep quality: A systematic review. Eur. J. Physiother..

[28] Kredlow M.A., Capozzoli M.C., Hearon B.A., Calkins A.W., Otto M.W. (2015). The effects of physical activity on sleep: A meta-analytic review. J. Behav. Med..

[29] Han K.S., Kim L., Shim I. (2012). Stress and Sleep Disorder. Exp. Neurobiol..

[30] Fang H., Tu S., Sheng J., Shao A. (2019). Depression in sleep disturbance: A review on a bidirectional relationship, mechanisms and treatment. J. Cell. Mol. Med..

[31] Papadimitriou G.N., Kerkhofs M., Kempenaers C., Mendlewicz J. (1988). EEG sleep studies in patients with generalized anxiety disorder. Psychiatry Res..

[32] Maes M., Song C., Lin A., De Jongh R., Van Gastel A., Kenis G., Bosmans E., De Meester I., Benoy I., Neels H. (1998). The effects of psychological stress on humans: Increased production of pro-inflammatory cytokines and th1-like response in stress-induced anxiety. Cytokine.

[33] Seo J.-S., Wei J., Qin L., Kim Y., Yan Z., Greengard P. (2016). Cellular and molecular basis for stress-induced depression. Mol. Psychiatry.

[34] Cooper M.L., Russell M., Frone M.R. (1990). Work Stress and Alcohol Effects: A Test of Stress-Induced Drinking. J. Health Soc. Behav..

[35] Chiu S.-I. (2014). The relationship between life stress and smartphone addiction on taiwanese university student: A mediation model of learning self-Efficacy and social self-Efficacy. Comput. Hum. Behav..

[36] Brand M., Wegmann E., Stark R., Müller A., Wölfling K., Robbins T.W., Potenza M.N. (2019). The Interaction of Person-Affect-Cognition-Execution (I-PACE) model for addictive behaviors: Update, generalization to addictive behaviors beyond internet-use disorders, and specification of the process character of addictive behaviors. Neurosci. Biobehav. Rev..

[37] Brand M., Young K.S., Laier C., Wölfling K., Potenza M.N. (2016). Integrating psychological and neurobiological considerations regarding the development and maintenance of specific Internet-use disorders: An Interaction of Person-Affect-Cognition-Execution (I-PACE) model. Neurosci. Biobehav. Rev..

[38] Sun X., Duan C., Niu G., Tian Y., Zhang Y. (2021). Mindfulness buffers the influence of stress on cue-induced craving for Internet among Chinese colleges with problematic Internet use. J. Behav. Addict..

[39] Elhai J.D., Dvorak R.D., Levine J.C., Hall B. (2017). Problematic smartphone use: A conceptual overview and systematic review of relations with anxiety and depression psychopathology. J. Affect. Disord..

[40] Ohmatsu S., Nakano H., Tominaga T., Terakawa Y., Murata T., Morioka S. (2014). Activation of the serotonergic system by pedaling exercise changes anterior cingulate cortex activity and improves negative emotion. Behav. Brain Res..

[41] Elhai J.D., Tiamiyu M.F., Weeks J.W., Levine J.C., Picard K.J., Hall B. (2018). Depression and emotion regulation predict objective smartphone use measured over one week. Pers. Individ. Differ..

[42] Wolniewicz C.A., Rozgonjuk D., Elhai J.D. (2019). Boredom proneness and fear of missing out mediate relations between depression and anxiety with problematic smartphone use. Hum. Behav. Emerg. Technol..

[43] Panicker J., Sachdev R. (2014). Relations among loneliness, depression, anxiety, stress and problematic internet use. Int. J. Res. Appl. Nat. Soc. Sci..

[44] Yen J.-Y., Lin H.-C., Chou W.-P., Liu T.-L., Ko C.-H. (2019). Associations among Resilience, Stress, Depression, and Internet Gaming Disorder in Young Adults. Int. J. Environ. Res. Public Health.

[45] China Internet Network Information Center (CNNIC) The 48th Statistical Report on China’s Internet Development. https://www.cnnic.com.cn/IDR/ReportDownloads/202111/P020211119394556095096.pdf.

[46] Yang H., Liu B., Fang J. (2021). Stress and Problematic Smartphone Use Severity: Smartphone Use Frequency and Fear of Missing Out as Mediators. Front. Psychiatry.

[47] Demirci K., Akgönül M., Akpinar A. (2015). Relationship of smartphone use severity with sleep quality, depression, and anxiety in university students. J. Behav. Addict..

[48] Exelmans L., Scott H. (2019). Social Media Use and Sleep Quality among Adults: The Role of Gender, Age and Social Media Checking Habit. https://www.researchgate.net/publication/332481174_Social_Media_Use_and_Sleep_Quality_among_Adults_The_Role_of_Gender_Age_and_Social_Media_Checking_Habit.

[49] Butt J., Weinberg R.S., Breckon J.D., Claytor R.P. (2011). Adolescent Physical Activity Participation and Motivational Determinants Across Gender, Age, and Race. J. Phys. Act. Health.

[50] Afifi M. (2007). Gender differences in mental health. Singap. Med. J..

[51] Brodaty H., Cullen B., Thompson C., Mitchell P., Parker G., Wilhelm K., Austin M.-P., Malhi G. (2005). Age and Gender in the Phenomenology of Depression. Am. J. Geriatr. Psychiatry.

[52] Aparicio-Martínez P., Ruiz-Rubio M., Perea-Moreno A.-J., Martínez-Jiménez M.P., Pagliari C., Redel-Macías M.D., Vaquero-Abellán M. (2020). Gender differences in the addiction to social networks in the Southern Spanish university students. Telemat. Inform..

[53] Zia A., Malik A.A. (2019). Usage of Social Media, Age, Introversion and Narcissism: A Correlational Study. Bahria J. Prof. Psychol..

[54] Krishnan V., A Collop N. (2006). Gender differences in sleep disorders. Curr. Opin. Pulm. Med..

[55] Roepke S.K., Ancoli-Israel S. (2010). Sleep disorders in the elderly. Indian J. Med. Res..

[56] Tsai P.-S., Wang S.-Y., Wang M.-Y., Su C.-T., Yang T.-T., Huang C.-J., Fang S.-C. (2005). Psychometric Evaluation of the Chinese Version of the Pittsburgh Sleep Quality Index (CPSQI) in Primary Insomnia and Control Subjects. Qual. Life Res..

[57] Leung D.Y., Lam T.H., Chan S.S. (2010). Three versions of Perceived Stress Scale: Validation in a sample of Chinese cardiac patients who smoke. BMC Public Heal..

[58] Wang W., Bian Q., Zhao Y., Li X., Wang W., Du J., Zhang G., Zhou Q., Zhao M. (2014). Reliability and validity of the Chinese version of the Patient Health Questionnaire (PHQ-9) in the general population. Gen. Hosp. Psychiatry.

[59] Yu N.X., Tam W., Wong P.T., Lam T.H., Stewart S.M. (2012). The Patient Health Questionnaire-9 for measuring depressive symptoms among the general population in Hong Kong. Compr. Psychiatry.

[60] Garabiles M.R., Lao C.K., Yip P., Chan E.W.W., Mordeno I., Hall B.J. (2020). Psychometric Validation of PHQ–9 and GAD–7 in Filipino Migrant Domestic Workers in Macao (SAR), China. J. Personal. Assess..

[61] Meng Y., Luo Y., Qin S., Xu C., Yue J., Nie M., Fan L. (2021). The effects of leisure time physical activity on depression among older women depend on intensity and frequency. J. Affect. Disord..

[62] Wang C.-J., Tsai H.-T., Tsai M.-T. (2014). Linking transformational leadership and employee creativity in the hospitality industry: The influences of creative role identity, creative self-efficacy, and job complexity. Tour. Manag..

[63] Anderson J.C., Gerbing D.W. (1988). Structural Equation Modeling in Practice: A Review and Recommended Two-Step Approach. Psychol. Bull..

[64] Walumbwa F.O., Orwa B., Wang P., Lawler J.J. (2005). Transformational leadership, organizational commitment, and job satisfaction: A comparative study of Kenyan and U.S. financial firms. Hum. Resour. Dev. Q..

[65] Hayes A.F. (2009). Beyond Baron and Kenny: Statistical Mediation Analysis in the New Millennium. Commun. Monogr..

[66] Yang M., Dijst M., Faber J., Helbich M. (2020). Using structural equation modeling to examine pathways between perceived residential green space and mental health among internal migrants in China. Environ. Res..

[67] Jansson-Fröjmark M., Lindblom K. (2008). A bidirectional relationship between anxiety and depression, and insomnia? A prospective study in the general population. J. Psychosom. Res..

[68] Stults-Kolehmainen M.A., Sinha R. (2014). The Effects of Stress on Physical Activity and Exercise. Sports Med..

[69] Burnham K.P., Anderson D.R. (2004). Multimodel inference: Understanding AIC and BIC in model selection. Sociol. Methods Res..

[70] Kardefelt-Winther D. (2014). A conceptual and methodological critique of internet addiction research: Towards a model of compensatory internet use. Comput. Hum. Behav..

[71] Brailovskaia J., Rohmann E., Bierhoff H.-W., Schillack H., Margraf J. (2019). The relationship between daily stress, social support and Facebook Addiction Disorder. Psychiatry Res..

[72] Gökçearslan Ş., Uluyol Ç., Şahin S. (2018). Smartphone addiction, cyberloafing, stress and social support among university students: A path analysis. Child. Youth Serv. Rev..

[73] Feng Y., Ma Y., Zhong Q. (2019). The Relationship Between Adolescents’ Stress and Internet Addiction: A Mediated-Moderation Model. Front. Psychol..

[74] Elhai J.D., Levine J.C., Hall B.J. (2019). The relationship between anxiety symptom severity and problematic smartphone use: A review of the literature and conceptual frameworks. J. Anxiety Disord..

[75] Saravanan C., Wilks R. (2014). Medical Students’ Experience of and Reaction to Stress: The Role of Depression and Anxiety. Sci. World J..

[76] Alvi T., Assad F., Ramzan M., Khan F.A. (2010). Depression, anxiety and their associated factors among medical students. J. Coll. Physicians Surg. Pak..

[77] Sreeramareddy C.T., Shankar P.R., Binu V.S., Mukhopadhyay C., Ray B., Menezes R.G. (2007). Psychological morbidity, sources of stress and coping strategies among undergraduate medical students of Nepal. BMC Med. Educ..

[78] Rogers L.Q., Courneya K.S., Oster R.A., Anton P.M., Robbs R.S., Forero A., McAuley E. (2017). Physical activity and sleep quality in breast cancer survivors: A randomized trial. Med. Sci. Sports Exerc..

[79] Rodriguez-Blanque R., Sánchez-García J., Sánchez-López A., Mur-Villar N., Aguilar-Cordero M. (2018). The influence of physical activity in water on sleep quality in pregnant women: A randomised trial. Women Birth.

[80] Bademli K., Lok N., Canbaz M., Lok S. (2019). Effects of Physical Activity Program on cognitive function and sleep quality in elderly with mild cognitive impairment: A randomized controlled trial. Perspect. Psychiatr. Care.

[81] Jackson E.M. (2013). Stress relief: The role of exercise in stress management. ACSM’s Health Fit. J..

[82] Esch T., Stefano G.B. (2010). Endogenous reward mechanisms and their importance in stress reduction, exercise and the brain. Arch. Med. Sci..

[83] Greenwood B.N., Fleshner M. (2011). Exercise, Stress Resistance, and Central Serotonergic Systems. Exerc. Sport Sci. Rev..

[84] Ghrouz A.K., Noohu M.M., Manzar D., Spence D.W., BaHammam A.S., Pandi-Perumal S.R. (2019). Physical activity and sleep quality in relation to mental health among college students. Sleep Breath..

[85] Wunsch K., Kasten N., Fuchs R. (2017). The effect of physical activity on sleep quality, well-being, and affect in academic stress periods. Nat. Sci. Sleep.

[86] Vandekerckhove M., Weiss R., Schotte C., Exadaktylos V., Haex B., Verbraecken J., Cluydts R. (2011). The role of presleep negative emotion in sleep physiology. Psychophysiology.

[87] Vandekerckhove M., Wang Y.-L. (2018). Emotion, emotion regulation and sleep: An intimate relationship. AIMS Neurosci..

[88] Deslandes A., Moraes H., Ferreira C., Veiga H., Silveira H., Mouta R., Pompeu F., Coutinho E.S.F., Laks J. (2009). Exercise and Mental Health: Many Reasons to Move. Neuropsychobiology.

[89] Macït H.B., Macït G., Güngör O. (2019). A research on social media addiction and dopamine driven feedback. Mehmet Akif Ersoy Üniversitesi İktisadi Ve İdari Bilimler Fakültesi Derg..

[90] Liu Q., Zhou Z.-K., Yang X.-J., Kong F.-C., Niu G.-F., Fan C.-Y. (2017). Mobile phone addiction and sleep quality among Chinese adolescents: A moderated mediation model. Comput. Hum. Behav..

[91] Lemola S., Perkinson-Gloor N., Brand S., Dewald-Kaufmann J.F., Grob A. (2015). Adolescents’ Electronic Media Use at Night, Sleep Disturbance, and Depressive Symptoms in the Smartphone Age. J. Youth Adolesc..

[92] Wood A.W., Loughran S.P., Stough C. (2006). Does evening exposure to mobile phone radiation affect subsequent melatonin production?. Int. J. Radiat. Biol..

