# Supplementary material for: Using Structural Equation Modeling to Examine Pathways between Physical Activity and Sleep Quality among Chinese TikTok Users
Source: Int J Environ Res Public Health. 2022 Apr 23;19(9):5142. doi: 10.3390/ijerph19095142 (PMC9105446; doi:10.3390/ijerph19095142)
Supplement: Supplementary file 1 [file ijerph-19-05142-s001.zip › ijerph-1662982-supplementary.pdf]

Supplemental Materials

Table S1. Model fits of the models.

Model	χ^2	df	p	χ^2/df	GFI	CFI	NFI	TLI	AGFI	IFI	RMSEA	AIC	BIC
M0	9.368	11	0.588	0.852	0.997	1.000	0.996	1.003	0.987	1.001	<0.001	77.368	230.105
M1 (Final)	21.870	23	0.528	0.951	0.993	1.000	0.990	1.001	0.986	1.001	<0.001	65.870	164.699
M2	42.321	23	0.008	1.840	0.986	0.991	0.980	0.986	0.973	0.991	0.036	86.321	185.150
M3	21.867	22	0.468	0.994	0.993	1.000	0.990	1.000	0.985	1.000	<0.001	67.867	171.188
M4	21.781	22	0.473	0.990	0.993	1.000	0.990	1.000	0.985	1.000	<0.001	67.781	171.102

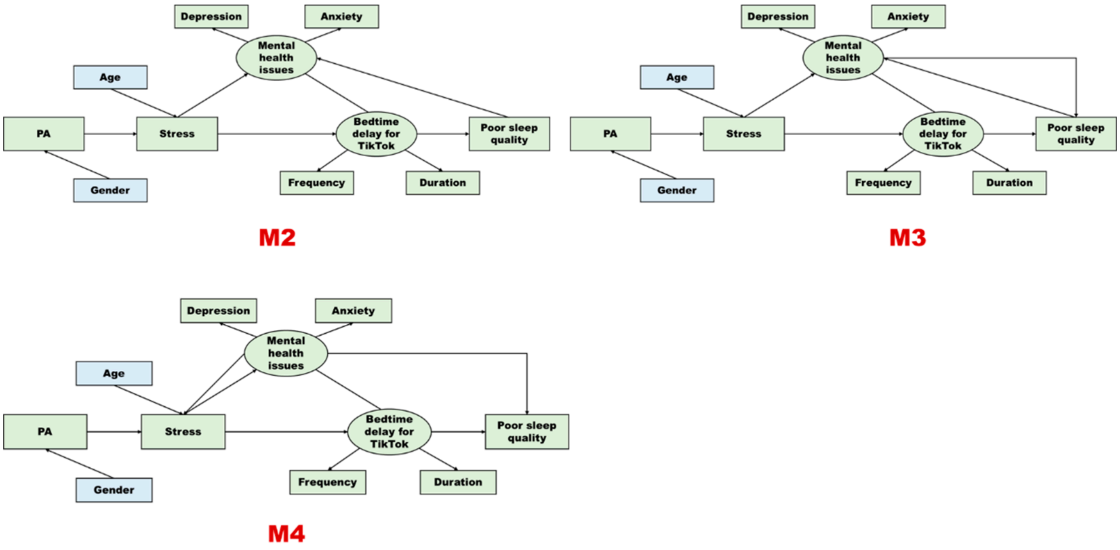

Figure S1. Structures of competing models.
